# Advances in IgA glycosylation and its correlation with diseases

**DOI:** 10.3389/fchem.2022.974854

**Published:** 2022-09-27

**Authors:** Li Ding, Xiangqin Chen, Hongwei Cheng, Tiantian Zhang, Zheng Li

**Affiliations:** Laboratory for Functional Glycomics, College of Life Sciences, Northwest University, Xi’an, China

**Keywords:** IgA, glycosylation, immunoglobulin, antibody, therapy

## Abstract

Immunoglobulin A (IgA) is the most abundant immunoglobulin synthesized in the human body. It has the highest concentration in the mucosa and is second only to IgG in serum. IgA plays an important role in mucosal immunity, and is the predominant antibody used to protect the mucosal surface from pathogens invasion and to maintain the homeostasis of intestinal flora. Moreover, The binding IgA to the FcαRI (Fc alpha Receptor I) in soluble or aggregated form can mediate anti- or pro- inflammatory responses, respectively. IgA is also known as one of the most heavily glycosylated antibodies among human immunoglobulins. The glycosylation of IgA has been shown to have a significant effect on its immune function. Variation in the glycoform of IgA is often the main characteration of autoimmune diseases such as IgA nephropathy (IgAN), IgA vasculitis (IgAV), systemic lupus erythematosus (SLE), and rheumatoid arthritis (RA). However, compared with the confirmed glycosylation function of IgG, the pathogenic mechanism of IgA glycosylation involved in related diseases is still unclear. This paper mainly summarizes the recent reports on IgA’s glycan structure, its function, its relationship with the occurrence and development of diseases, and the potential application of glycoengineered IgA in clinical antibody therapeutics, in order to provide a potential reference for future research in this field.

## Introduction

IgA is the most abundant immunoglobulin synthesized in the human body, with a yield of approximately 66 mg kg^−1^ day^−1^, more than the total amount of all other types of immunoglobulins (Yoo and Morrison, 2005). IgA mainly exists at mucosal surfaces, providing the first line of immune defense against pathogens invasion. Moreover, IgA is also an important serum immunoglobulin second only to IgG, which can mediate anti- or pro- inflammatory responses by binding to the FcαRI in soluble or aggregrate form, respectively. As the main immunoglobulins in the human body, both IgG and IgA are glycosylated. By far the glycan function of IgG has been confirmed (Cobb, 2020), but the research on IgA glycosylation and its involvement in related diseases is still in early stages.

IgA is known as one of the most heavily glycosylated glycoproteins among human immunoglobulins. Unlike IgG, which contains only one conserved *N*-glycosylation site, both IgA1 and IgA2 monomers contain multiple *N*-glycosylation sites, and IgA1 has another nine potential *O*-glycosylation sites in the hinge region. The number of these glycosylation sites and the composition and structure of glycans have been reported to be considerably heterogeneous in individuals (Novak et al., 2000; Takahashi et al., 2012). This can affect the recognition and binding of IgA to pathogens and its neutralization to pathogenic microorganisms. Moverover, variation in IgA glycosylation has been reported to be closely associated with the occurrence and development of many diseases. For example, the aberrantly glycosylated IgA has been found in the serum of patients with ovarian cancer, breast cancer, colorectal cancer, hepatitis B virus-related liver cancer (Ruhaak et al., 2016; Lomax-Browne et al., 2019; Zhang et al., 2019). Furthermore, the abnormal glycosylation of IgA is often the main characterization of severe autoimmune diseases (Singh et al., 2014), especially IgA nephropathy (IgAN) and IgA vasculitis (IgAV) (Suzuki et al., 2018; Suzuki and Novak, 2021). Therefore, the aberrant glycan of IgA is expected to be an important indicator for clinical diagnosis. Since the relationship between IgA glycosylation and nephropathy is being widely demonstrated, IgA glycosylation has long been the focus of immunology research and exploration of antibody therapeutics.

## Structure, function, and classification of immunoglobulin A

**FIGURE 1:**
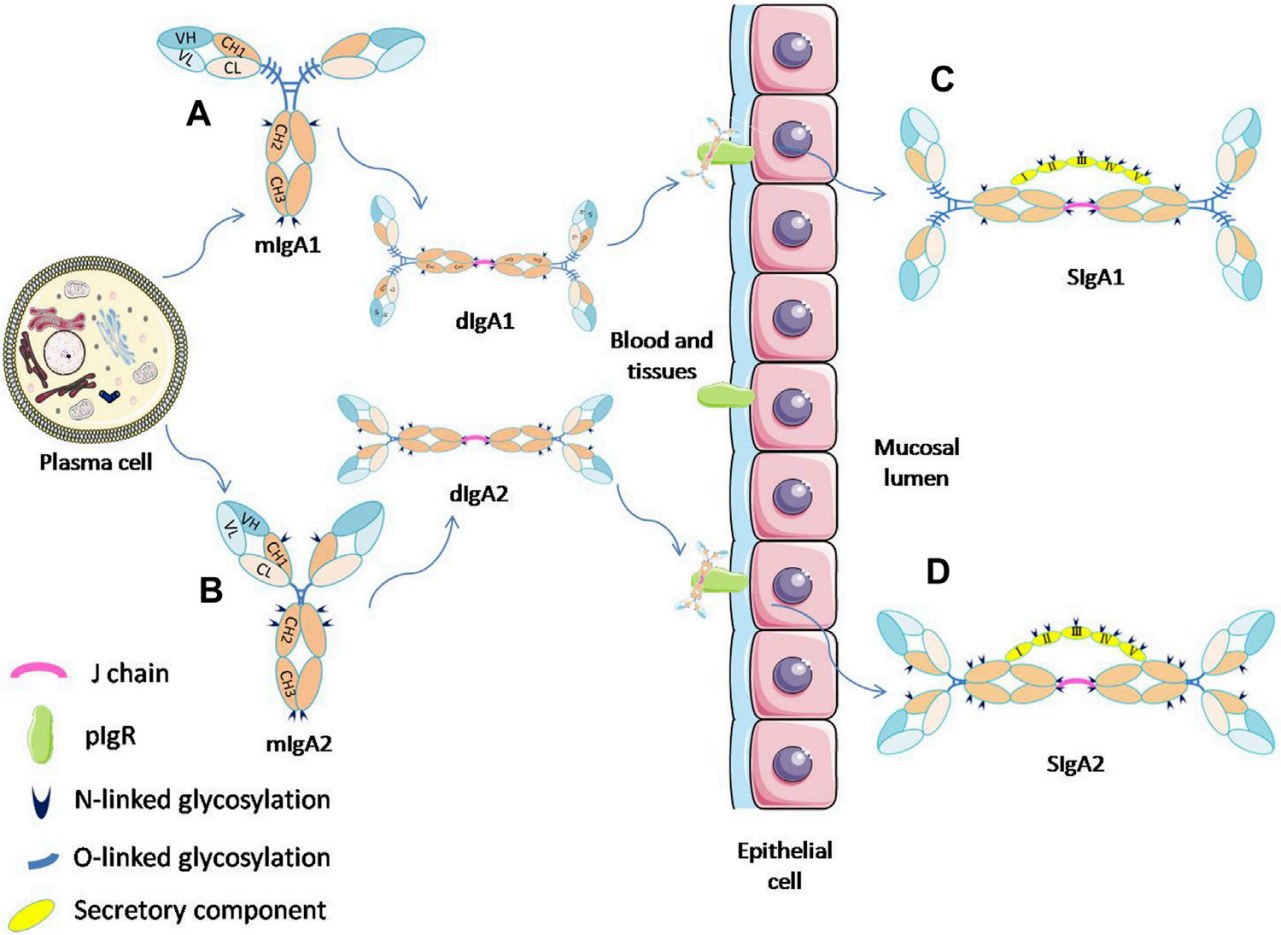
Schematic diagram of the process of SIgA formation by mIgA and its glycosylation sites. IgA monomers **(A,B)** present in the mucosal plasma cells can form dimmers through the J-chain. The dIgA is then transported across the epithelium by binding to pIgR. At the luminal side, dIgA is released from the pIgR, and a part of the receptor (SC) remains attached to dIgA to form SIgA **(C,D)**. (Davis et al., 2020; van Gool and van Egmond, 2020).

IgA is an immunoglobulin found in all categories of mammals and birds. It is a heterodimeric protein composed of two heavy chains (H chain) and two light chains (L chain) according to the genetic sequence analysis and functional comparison (Kawamura et al., 1992; Woof and Russell, 2011). The IgA molecule can be folded into different globular domains, including four heavy chain domains (VH, CH1, CH2, and CH3) and two light chain domains (VL and CL) (Figures 1A,B). Functionally, IgA can be divided into the variable domain responsible for binding to an antigen (Fab segment) and the constant domain that is important for binding to the Fc receptor (Fc segment). The constant domains of CH1 and CH2 are linked by an 8–21 amino acid sequence called the hinge region. In addition, there is a conserved tail structure consisting of 18 amino acids at the C-terminal of the CH3 region on the IgA heavy chain. This kind of tail is essential for the formation of divalent and multivalent structures of IgA (Xie et al., 2021).

In the human body, IgA can exist in a variety of forms. The monomeric IgA (mIgA), which is mainly present in serum, is produced by plasma cells or marginal zone B cells in bone marrow, spleen and lymph nodes. By contrast, the secretory IgA (SIgA) on mucosal surfaces or in external secretions usually appears in the form of dimer or polymer with a high molecular weight, and it is originaly produced by local plasma cells near epithelium. IgA can bind to the specific receptor FcαRI (CD89) on the surface of neutrophils, monocytes, and macrophages. The interation of IgA with FcαRI can trigger the corresponding inflammatory response, and play an important role in the mediation of phagocytosis, superoxide production, cytokine release, etc. (Woof and Russell, 2011; Pan et al., 2021).

### Serum immunoglobulin A

The human monomeric IgA can be divided into two subtypes, IgA1 and IgA2, based on the structure (Figures 1A,B). Although IgA presents in all catergories of mammals, there are notable species difference. Most mammals (including mice and rats which are widely used as animal models) only have a single subclass of IgA that resembles human IgA2, while rabbits and other lagomorphs have up to 15 subclasses of IgA. Humans and related primates, such as chimpanzees, gorillas, and gibbons, are the few species possessing both of IgA1 and IgA2 subtypes (Woof and Russell, 2011; Pan et al., 2021). The IgA in human serum usually appears as a monomer, mainly of the IgA1 subtypes (about 84% are IgA1 and 16% are IgA2). However, a small amount of dimeric or polymeric IgA can also be found. The amino acid sequences of IgA1 and IgA2 have high similarity, and the structural differences between them mainly exist in the length and glycosylation site in the hinge region. IgA1 has a longer hinge region (13 amino acids more than IgA2) consisting of two repeated amino acid sequences. These sequences are rich in *O*-glycosylation sites of serine and threonine, which can be highly sialylated. However, IgA2 lacks the hinge region with the *O*-glycosylation site, and has only *N*-glycosylation sites and a lower level of sialylation (Yoo and Morrison, 2005; Takahashi et al., 2012). The hinge region of IgA1 with *O*-glycosylation helps to improve its recognition to antigens, but makes it more susceptible to being hydrolyzed by bacterial proteases (Senior and Woof, 2005a).

Recent studies have shown that serum IgA unconjugated with an antigen can modulate immune effect by inhibitory ITAM (Immunoreceptor tyrosine-based activation motif) signaling, and promote anti-inflammation response, which is important to maintain the stability of the internal environment of the human body (Leong and Ding, 2014; van Gool and van Egmond, 2020). For example, the binding of serum monomeric IgA to FcαRI can inhibit the oxidative respiratory burst, and the IgG-mediated phagocytosis and production of cytokine (Lecocq et al., 2013; Saha et al., 2017). The binding of serum IgA to FcαRI can also inhibit the IgE-mediated asthma in transgenic mice (Pasquier et al., 2005). By this kind of FcαRI-dependent ITAMi (inhibitory ITAM) signaling pathway, the serum IgA can maintain homeostasis and resist the receptors of FcγR or FcεRI mediated cellular activities during inflammation or allergy (Kanamaru et al., 2008).

### Secretory immunoglobulin A

SIgA is the main immunoglobulin present on mucosal surfaces and in external secretions such as saliva, milk, and respiratory and gastrointestinal secretions (Corthesy, 2013). SIgA can be divided into two subtypes, SIgA1 and SIgA2. These two subtypes have different proportions in different tissues. For example, IgA1 and IgA2 account for 65% and 35% in saliva and 39% and 61% in the colostrum, respectively. SIgA is produced by the coordination of two different types of cells in the human body (Figures 1C,D). The dimer IgA (dIgA) with attached J chain is produced by plasma cells close to the epithlium. It can bind to the polymeric immunoglobulin receptor (pIgR) expressed on the basolateral membrane of epithelial cells, and then be transported across the epithelium to the luminal side. At the luminal side, dIgA is released by cleavage from the transmembrane tail of pIgR. The ectodomain of pIgR, referred to as secretory component (SC), remains attached to the IgA molecule by binding with both Fc-tails and J-chain, thereby forming SIgA (Wang et al., 2020).

The mucosal system is the first line of human immune defense against pathogens and harmful substances. As the main immunoglobulin appears on mucosal surfaces, SIgA plays an incredible role in maintaining mucosal homeostasis (Corthesy, 2013). It can inhibit toxins or viruses passing through the epithelial layer and prevent the colonization and invasion of pathogenic bacteria (Rollenske et al., 2021; Pabst and Izcue, 2022). In addition, the conformational changes of SIgA after binding with antigens facilitate the binding of this SIgA immune complex to FcαRI, which can trigger further adaptive immune response (Geuking et al., 2012).

## Structure of immunoglobulin A glycosylation

Glycosylation is one of the most common and diverse post-translational modification of proteins and always has a profound functional effect on the conjugated proteins. The hemiacetal hydroxyl group of glycans can condense with the side chain amino group of Asparagine (Asn) or the hydroxyl group of Serine (Ser)/Threonine (Thr) residues on the protein sequence to form *N*- or *O*-linked protein glycosylation (Reily et al., 2019). Among immunoglobulins, both *N*-glycosylation and *O*-glycosylation have been widely studied. The *N*-glycans are highly heterogeneous, but all of them contain a core structure composed of two *N*-acetylglucosamine (GlcNAc) and three mannose (Man) residues. The core structures are synthesized in the rough endoplasmic reticulum. It can be modified and further extended to Glc3Man9GlcNAc2. Then the structure is attached onto Asn residue in nascent protein. After being transported to the Golgi, they can be modified under various glycosyltransferases and finally processed to the high mannose, hybrid, or complex *N*-glycans. The *N*-glycosylations are common on plasma membranes and secretory glycoproteins, including immunoglobulins such as IgA and IgG, as well as proteins on the cell surface (de Haan et al., 2020).

The *O*-glycosylation can be divided into different types according to the first monosaccharide attaching to the Thr or Ser residue on the protein sequence, such as *O*-linked galactose (Gal) or *N*-acetylgalactosamine (GalNAc), *O*-linked glucose (Glc) or GlcNAc, and *O*-linked mannose (Man). Unlike *N*-glycosylation, all of these *O*-glycans are synthesized by the stepwise addition of a single monosaccharide to proteins after translation. The most common *O*-glycosylation in human belongs to the mucin-type, and the basic structure of which is R-GalNAc-α1- Ser/Thr. The *O*-GalNAc can be extended by various glycosyltransferases in the Golgi and assembled into different kinds of *O*-glycans. The mucin-type *O*-glycans include eight core structures based on the second monosaccharide added and are usually terminated with the monosaccharide of salic acid (Sia, SA) or fucose (Fuc). These mucin-type glycans are rich in the secretes of the digestive tract, respiratory tract, and reproductive tract. Thus forming a viscous barrier between microorganisms and mammalian hosts. According to current studies, *O*-glycosylations in the hinge region of IgA are also dominated by these mucin-type glycans (Takahashi et al., 2012; Lehoux and Ju, 2017).

IgA is heavily glycosylated with both *O*- and *N*-glycans. The composition and structure of these glycans have been shown to be heterogeneous according to different individuals and in different tissues, and can vary at different pathological and physiological stages. IgA glycosylation is critical for its biological function, including bacterial attachment, pathogen clearances, and immunoregulation.

### O-glycosylations on the hinge region of immunoglobulin A

**FIGURE 2:**
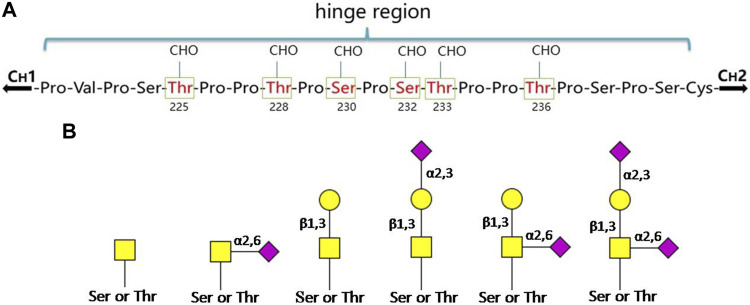
The O-glycosylation sites and glycan structures in human serum IgA1. **(A)** Boxed and numbered amino acids indicate the sites of attachment of O-linked glycans (CHO: carbohydrate) in the hinge region. **(B)** The core structures of O-glycans on human IgA1 are composed of GalNAc or GalNAcβ-1,3-Gal. Both of them can be modificated by Sia in different linkages (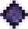: GalNAc; 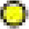: Gal; 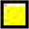: Sia) (Novak et al., 2000; Novak et al., 2018; Hansen et al., 2021).

There are two isotypes of IgA, IgA1 and IgA2, and IgA2 can be further divided into three allotypes: IgA2m (1), IgA2m (2) and IgA2 (n). IgA1 has nine potential *O*-glycosylation sites (containing Ser or Thr residues) in the hinge region, of which the sites of Thr225, Thr228, Ser230, Ser232, Thr233 and Thr236 have been confirmed (Figure 2A). The Thr225 and Thr236 residues are not always occupied as that found in human serum IgA1. The monosaccharide composition of the *O*-glycans in the hinge region is variable, mainly including GalNAc, Gal and different forms of Sia (Xue et al., 2013; Lehoux and Ju, 2017). Usually, these *O*-glycan structures are of the Core-1 type (Lehoux and Ju, 2017), in the form of T antigen (Gal-β-1,3-GalNAc-α1- Ser/Thr), sialylated T or disialylated T antigens. Sialylation can occur either on β-1,3-Gal or α1-GalNAc through α-2,3 or α-2,6 linkage (Figure 2B). Among them, the most abundant is the core 1 mono-sialylated T antigen (Neu5Acα2–3Galβ1–3GalNAc) (Takahashi et al., 2012). The truncated *O-*glycans, Tn (GalNAc-α1-Ser/Thr) and/or sialylated Tn (STn) were also found to distribute in a minor IgA1 fraction. Compared to the simple Core-I structures of serum IgA1, the SIgA1 *O-*glycans are much larger and more elaborate. Besides the Gal β-1,3 Core-I *O-*glycans, the Gal β-1,4 Core-II and its lactosamine extension with/without α-2,3 sialylation or α-1,4 fucosylation have also been found on SIgA1. Moreover, the *O-*glycans of SIgA1 vary in size widely from 2 to 15 monosaccharide residues, and more than 50 glycans structures have already been identified (Royle et al., 2003). The range and distribution of different *O-*glycan isomers at the sites of hinge region of IgA1 create a complex heterogeneity of surface epitopes.

### *N*-glycosylations on the heavy chain of immunoglobulin A

In addition to the *O*-glycosylation reported in the hinge region, there are two other *N*-glycosylation sites on the IgA1 heavy chain, which is the N144 in the CH2 region and the N340 in the CH3 region. Compared to IgA1, IgA2 has a shorter hinge region and lacks the corresponding *O*-glycosylation site, but each allotype contains four *N*-glycosylation sites (N131, N327, N47, N205), and IgA2m (2) and IgA2 (n) also have a fifth *N*-linked glycan in the CH1 region (N92) (Steffen et al., 2020). The sites of N144 and N340 on IgA1 are identical to the N131 and N327 sites of IgA2, respectively, in amino acid sequences.

The *N*-glycosylation of IgA1 and IgA2 have been widely elucidated (Royle et al., 2003; Deshpande et al., 2010; Huang et al., 2015; Steffen et al., 2020; Huus et al., 2021; Suzuki and Novak, 2021). Sixteen *N*-glycan structures have already been identified on five glycosylation sites (N47, N92, N144/N131, N205, and N340/N327) on IgA heavy chains (Huang et al., 2015). As shown in Table 1, most of the *N*-glycans on IgA are biantennary structures, and a few of them are tri- or tetra antennary structures and high mannose types. In contrast to the orientation of IgG glycans towards the interior of both Cγ2 domains, molecular modeling revealed that IgA glycans were oriented towards the exterior of the molecule. This external oriention could make the IgA *N*-glycans more accessible to the glycosyltransferases in local cells, and cause the cell-specific glycoforms and extensive glycan heterogeneity (Mattu et al., 1998). Great differences have also been reported in the structures of glycans between serum IgA1 and IgA2. Comparing the *N*-glycosylations at the two conserved sites of N144 and N340 in IgA1 with those of N131 and N327 in IgA2, it was found that there is relatively little sialylation, galactosylation, or fucosylation on the IgA2 subtype. The *N*-glycan structures in IgA2 are relatively simple and mostly high mannose or biantennary type, while for IgA1, it expresses a high level of sialylation and galactosylation, not only on the glycans of *N*-glycosylation sites but also on the glycans of *O*-glycosylation sites in the IgA1 hinge region. It has been reported that about 80% of the glycans on IgA1 are glycosylated with Gal and more than 75% of them are glycosylated with terminal Sia (Lehoux and Ju, 2017).

**TABLE 1:** The N-glycosylation sites and glycan structures on the heavy chain, J chain, and SC of IgA^a^.

Name	Glycosylation site	Glycan structure
IgA heavy chain IgA heavily chains	N_47_	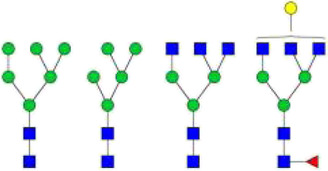
	N_92_	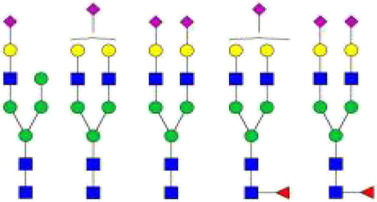
	N_144_/N_131_	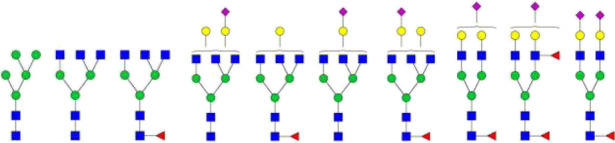
	N_205_	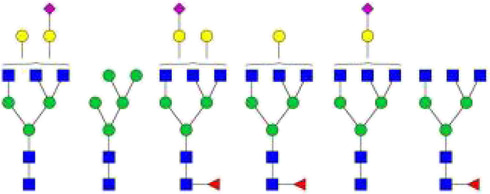
	N_340_/N_327_	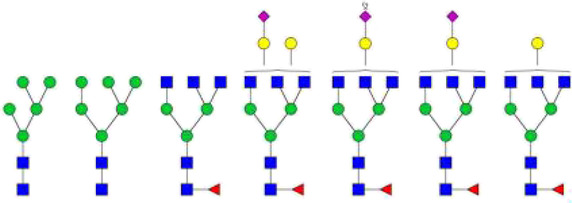
J chain	N_71_	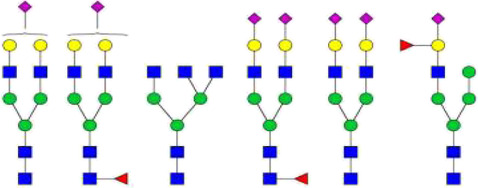
SC	N_83_	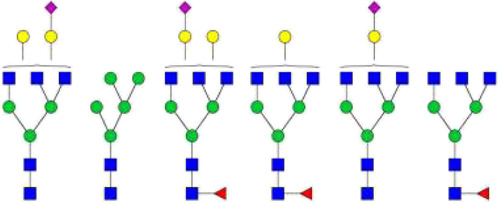
	N_90_	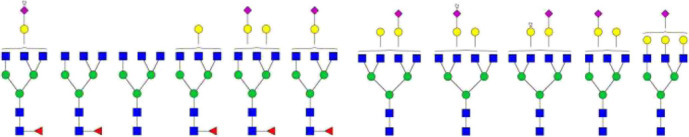
		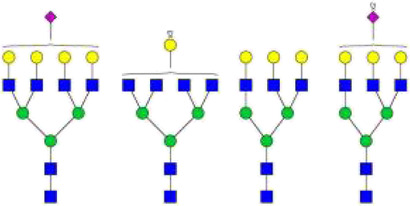
	N_135_	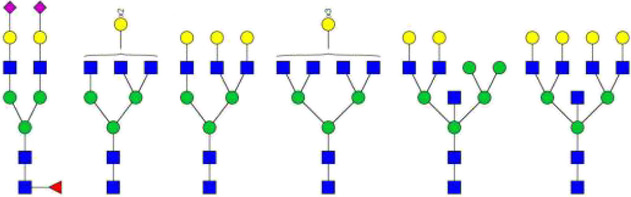
	N_186_	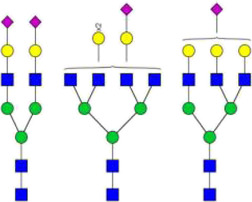
	N_421_	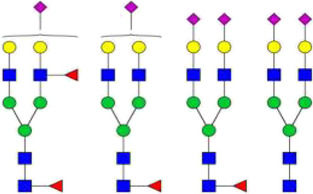
	N_469_	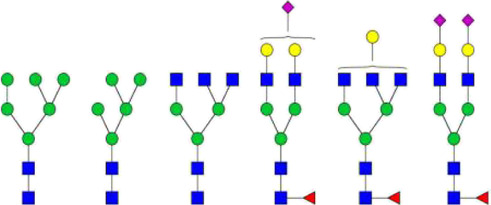
	N_499_	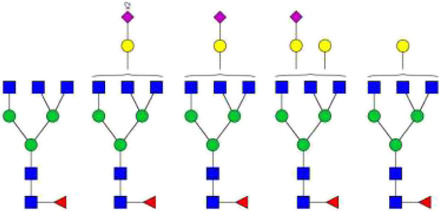

a The glycan structures were drawn based on the previous report (Huang et al., 2015).

(Notes: 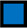, GlcNAc; 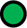, Mannose; 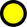, Gal; 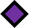, Sia; 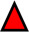, fucose).

### Glycosylations on the J chain and secretory component of secretory immunoglobulin A

SIgA is generally a dimeric complex composed of two identical mIgA, covalently bound together with J-chains (a molecular weight of about 16 kD) and SC (a molecular weight of about 80 kD). Among them, the J chain has one potential *N*-glycosylation site and the SC has seven potential *N*-glycosylation sites, and the *N*-glycans at these sites are mainly biantennary structures terminated with GlcNAc or Sia. Up to now, more than 30 *N*-glycan structures at seven glycosylation sites (N83, N90, N135, N186, N421, N469, and N499) of SC and six *N*-glycan structures at N71 of the J-chain have been identified (Royle et al., 2003; Deshpande et al., 2010; Huang et al., 2015). As shown in Table 1, almost all of the glycans found at N71 of the J chain are glycosylated with one or two terminal Sia. Half of the glycans found at this site are core-fucosylated (Huang et al., 2015). The *N*-glycans at this site are required for the correct conformation of J chain, deletion of them can prevent the dimeric IgA formation and inhibit the pIgR-mediated epithelial transport of IgA (Xie et al., 2021).

The SC of SIgA expresses a wide range of glycan epitopes. The majority of them are glycotosylated, nonbisected structures (Table 1). It has been reported that over 75% of these *N*-glycans are sialylated, and most of the terminal Sia are α-2,6 linked to Gal. Over 65% of the structures contain core fucose (Royle et al., 2003). Usually, the *N*-glycans on the IgA heavy chain are masked by the SC. However, the non-covalent interaction of SC with SIgA heavy chain could be disrupted by the binding of SC *N*-glycans to bacterial adhesins or cellular lectins. The exposed high mannose oligosaccharides on the heavy chain would be recognized by soluble MBL (mannose-binding lectins) or other mannose receptors on macrophages and dendritic cells, and subsequently promote the adaptive immune system (Royle et al., 2003).

## Effect of immunoglobulin A glycosylation on its biological function

### Effect of immunoglobulin A glycosylation on the immune regulation of FcαRI

IgA can bind to various receptors to exert different biological functions, such as FcαRI (CD49), Fcα/μR (CD351), pIgR, transferrin receptor (CD71), asialoglycoprotein receptor (ASGPR), and FcRL4 (CD307d), of which only FcαRI and FcRL4 are IgA-specific recognition receptors. FcαRI can bind to both IgA1 and IgA2, while FcRL4 usually binds to polymeric IgA1 or IgA1 in immune complexes (but not to secretory IgA1) (Breedveld and van Egmond, 2019; Monteiro et al., 2020; Van De Winkel, 2003). As the most important and specific recognition receptor of IgA, FcαRI is mainly expressed by myeloid cells, such as monocytes, neutrophils, macrophages and dendritic cells. The interation of IgA with specific FcαRI can mediate cell degranulation, phagocytosis, chemotaxis, and antibody-dependent cytotoxicity. Both IgA and the FcαRI receptor are glycosylated proteins. Current research has suggested that the *N*-glycan on IgA can affect its thermal stability but may not affect its binding ability to the receptor (Gomes et al., 2008), whereas the *N*-glycan on FcαRI can significantly affect the affinity between IgA and FcαRI (Göritzer et al., 2019). However, by the binding of IgA1 to FcαRI, conformational changes can be induced in the highly *O*-glycosylated hinge region of IgA1 through a long-distance electrostatic effect (Posgai et al., 2018). This kind of conformational change will make the terminal galactose-deficient *O*-GalNAc-linked glycans, which usually appear on the IgA at pathological states, more easily recognized by its specific lectin HAA, transferrin receptors, and other autoantibodies on the surface of lymphocytes cells, and eventually cause the formation of immunoprecipitation complexes in the glomerular mesangial region of IgAN patients (Suzuki and Novak, 2021).

The binding of IgA-FcαRI can often active the pattern recognition receptors (such as Toll-like receptors), thereby inducing the expression of cytokines in antigen presenting cells, which is the basis for regulating inflammatory response, triggering innate immunity and adaptive immunity. Different kinds of inflammatory responses can be induced depending on the combination states of IgA with FcαRI (Breedveld and van Egmond, 2019). In normal physiological state, serum IgA mainly exists in soluble monomeric form. The affinity of monomeric IgA (not complex to an antigen) to FcαRI is low. Their combination can initiate inhibitory ITAM (ITAMi) signaling, resulting in anti-inflammatory immune response. In this way, serum IgA plays an important role in maintaining the internal homeostasis and avoiding the occurrence of some undesired persistent inflammatory phenomena (Mkaddem et al., 2014). But in pathological conditions, the polymeric IgA or IgA complex increases. These kind of aggregated IgA can cross-link with the FcαRI receptor on the surface of myeloid cells to active ITAM signaling, leading to pro-inflammatory immune response (Hansen et al., 2019). In this way, the IgA complex (IgA-opsonized antigens) can recruit and activate FcaRI expressing immune cells (especially for neutrophils) to eliminate pathogens and tumors cell, which is important in controlling infections (Brandsma et al., 2019; Davis et al., 2020). In the serum of patients such as IgAN, the aberrant glycoform of IgA1 can cause a great increase in IgA immune complexes. The excessive IgA immune complexes will make the FcαRI-mediated immune regulation to become out of control. And the persistent pro-inflammatory responses will result in severe tissue damage that can be observed during the process of chronic inflammation and autoimmunity diseases.

It was also reported that the effector functions of IgA on myeloid cells depend on its subclasses and glycosylation. Steffen et al. have compared the immune effects of IgA1 and IgA2 on neutrophils and macrophages. In the study, IgA antibodies were heat aggregated or immobilized to mimics immune complexes. Under these conditions, it was found that the lower sialylated IgA2 can effectively induce pro-inflammatory responses like FcαRI-dependent NET (neutrophil extracellular traps) fromation and cytokine production, whereas IgA1 with higher sialylation level does not have pronounced effect. Enzymatic removal of Sia or the whole *N*-glycans can significantly increase the pro-inflammatory capacity of IgA1 to even the same level as IgA2 (Steffen et al., 2020). In patients with autoimmune diseases such as rheumatoid arthritis, the disease-specific autoantibodies was found to shift toward the pro-inflammatory IgA2 subclass. And the proportion of IgA1 and IgA2 autoantibodies is significantly related to the progression of the disease (Steffen et al., 2020). IgA may balance the corresponding functional effects in human body through this way, so a deeper understanding of the function of IgA subtypes and their different glycosylations will help to reveal the dual role of IgA as a tolerance and inflammatory inducer.

### Effect of immunoglobulin A glycosylation on its half-life

Previous studies have shown that IgA1 and IgA2 have different pharmacokinetic characteristics, which is determined by their different glycosylation (Rifai et al., 2000). IgA2 is mainly cleared in the liver through the receptor of ASGPR that recognizes terminal Gal residues on the glycans, and the clearance of IgA in this pathway can be significantly inhibited by injection of excess Gal-containing ligands or in ASGR-deficient mice. In contrast, only a small part of IgA1 is cleared through this pathway because of its highly *O*-glycosylated hinge region. It was reported that IgA1 lacking the hing region can be cleared more rapidly compared to the wild-type (Rifai et al., 2000). The efficient clearance of IgA2 in the liver, rather than IgA1, can partly explain why the serum level of IgA1 is much higher than IgA2.

In the mucosal system, IgA2 is more stable than IgA1 due to the lack of a highly *O*-glycosylated hinge region that is susceptible to the protease in bacteria (Senior and Woof, 2005b). Compared with mIgA, SIgA has the SC surrounded, which makes the entire molecule of the antibody to have a higher stability. Moreover, the glycans on the SC can effectively prevent the degradation of IgA by the protease in bacteria. Therefore, the half-life of SIgA on the mucosal surface is much longer, usually three times as that of IgG, and its protective effect in the human exocrine tract can last more than 4 months (Corthesy, 2013).

### Effect of immunoglobulin A glycosylation on the complement system activation

The role of IgA in the activation of the complement system is still not clear. It is generally believed that IgA cannot activate the complement system through the classical pathway, however, in some cases, it can activate the complement system through the alternative pathway or lectin pathway (Medjeral-Thomas et al., 2021). It was reported that the overexpressed galactose-deficient IgA1 (Gd-IgA1) can bind to mannose-binding lectin (MBL) in patients with IgA nephropathy (IgAN) (Chiu et al., 2021). After treatment with galactosidase, the binding abilities of monovalent and multivalent IgA1 and IgA2 to MBL can be improved by more than 10-fold. It is supposed that this may happen due to the exposure of high mannose structures at the site such as Asn340/327 after removal of the complex N-glycan (Terai et al., 2006). In IgAN patients, Gd-IgA1 mainly exists in the polymeric or immune complex status. This kind of IgA1 can recognize MBL and activate the complement system, resulting in the deposition of component C4 in the mesangial membrane (Medrano et al., 2022). Meanwhile, the anti-Gd-IgA1 IgG in the immune complex will further activate the C3 component in the complement system (Chen et al., 2019). Additionally, Sia was also reported to play an important role in the recognition and binding of IgA1 and IgA2 to the complement C3b components (Nikolova et al., 1994).

### Anti-viral effector mechanisms of immunoglobulin A glycosylation

It has been shown from previous studies that the antiviral effect of IgA is much greater than that of IgG, which may be related to its high level of sialylation on the glycochains. In contrast to the fact that only 10% of *N*-glycans are modified with Sia in IgG, more than 90% of the *N*-glycans are sialylated in IgA. Muramatsu et al. (2014), Maurer et al. (2018) have compared the *in vitro* anti-influenza ability between each isotype of the monoclonal IgA and IgG antibodies. Maurer et al. (2018) found that the neutralization of IgA to the influenza virus was 10–1,000 times higher than that of IgG, and its antiviral activity was mainly mediated by the α2,3- and α2,6-linked Sia modification of the complex *N*-glycans at the C-terminal tail of IgA. The sialosides at the tailpiece *N*-glycosylation site can specifically bind to the neuraminidases of virions, thereby interfering with the attachment of the influenza A virus (IVA) as well as other enveloped viruses using Sia as receptors to the host cell surface (Maurer et al., 2018). Muramatsu et al. (2014) showed that IgA can effectively inhibit the extracellular release of the influenza virus and keep them aggregated on the surface of the infected cells because of its multivalency, thus making the anti-influenza ability of IgA significantly higher than IgG. Moreover, in comparision to the monoclonal anti-hemagglutinin (HA) IgG that was only specific to a certain subtype of IVA, the anti-HA IgA showed higher cross-binding activities against different subtypes of IVA. At present, the effect of the inactivated influenza virus vaccine mainly relies on the production of specific IgG antibodies in serum, which can only target a class of virus with similar antigens. However, the heterosubtypic immunity of IgA enables the human body to obtain cross-protection against different influenza virus subtypes (Muramatsu et al., 2014; Suzuki et al., 2015), so it is expected to be applied in the development of new influenza vaccines in the future (van Riet et al., 2012). In addition to IVA, IgA has also been shown to play an important role in neutralizing rotavirus, human immunodeficiency virus (HIV), novel coronaviruses, etc. (Feng et al., 2002; Paul et al., 2014; Ejemel et al., 2020). Sun et al. (2021) reported recently that the monoclonal antibody of IgA1 expressed by the glycoengineered plants of *Nicotiana benthamiana* exhibited a significantly higher activity of neutralization against syndrome coronavirus 2 (SARS-CoV-2) compared to IgG1. Furthermore, it was reported that the neutralization potency of the glycosylated IgA1 dimer, compared to the corresponding monomer, can be increased by up to 240-fold (Sun et al., 2021).

### Regulation of immunoglobulin A glycosylation on the intestinal microorganisms

As the most abundant antibody present at the mucosal surface, SIgA plays a prominent role in the host-pathogen defense of the mucosal system (Geuking et al., 2012; Rollenske et al., 2021; Pabst and Izcue, 2022). SIgA can protect the intestinal mucosal barrier by regulating the composition of microorganisms, and the glycans of SIgA is crucial for its interaction with the intestinal microbiota and defense against the potential attack of pathogens (Mathias and Corthésy, 2011b; Nakajima et al., 2018). The *N*-glycans on the SC of SIgA can adhere to the mucosal epithelium so that it can be neatly arranged on the mucosal surface, forming an isolated protective layer that effectively prevents the invasion of pathogens (Perrier et al., 2006; Mathias and Corthésy, 2011a). Besides, it was reported that the *N*-glycans on the SC of SIgA can bind with the peptidoglycans (PNG) on the Gram-positive cell wall directly. Removal of these *N*-glycans could dramatically reduce the binding of SIgA to Gram-positive bacteria (Mathias and Corthésy, 2011a; Mathias and Corthésy, 2011b). Other studies showed that IgA glycans can also interact with the lipopolysaccharide (LPS) of Gram-negative bacteria such as *Bacteroides thetaiotaomicron* (*B. theta*). The glycan-LPS interactions facilitate the colonization by the microbiome and promote the regulation of the intestinal flora by improving the gene expression profiles for polysaccharide utilization and butyrate production (Donaldson et al., 2018; Nakajima et al., 2018). More importantly, the *O*-glycans in the hinge region and the *N*-glycans on the heavy chains of SIgA contain structures of carbohydrate epitopes that can bind to the adhesins of bacteria or be used as competitive inhibitors of pathogens, such as Galβ1-4/3GlcNAc, Fucα1-3/4GlcNAc, and α2-3/6-linked Sia. These carbohydrate epitopes provide additional binding sites against bacteria or other pathogens independent of the four antigen sites on the Fab of SIgA, thus effectively preventing their adhesion to epithelial cells (Royle et al., 2003).

Due to the diversity of SIgA glycosylation and its importance in the protection of epithelial cells from pathogens invasion, it has the potential to be explored as a therapeutics for mucosal immune diseases. For example, Balu et al. (2011) constructed a novel human IgA1 using a single-chain variable fragment clone and inoculated a purified monomeric IgA1 containing both *O*- and *N*-linked glycosylations into human CD89 transgenic mice, and they found that the inoculated mice had a high ability to resist *Mycobacterium tuberculosis* infection. Vidarsson et al. (2001) produced a unique human IgA chimeric antibody in hamster kidney cells and found that it plays an important role in preventing meningococcal infection. In addition, it has been demonstrated that the specific binding between the highly sialylated glycans of SIgA in human milk and pathogens can provide passive immunity to newborns, and protect them from the fatal infections, such as *Streptococcus pneumoniae* and S-fimbriated *Escherichia coli* (Schroten et al., 1998; Goonatilleke et al., 2019).

## Abnormal of immunoglobulin A glycosylation with disease development

Studies are increasingly showing that protein glycosylations are involved in many immune processes, including immune cell differentiation, cell-cell recognition, signal transduction, cell activation and adhesion, the secretion of immune molecules, and immune enhancement and suppression. Antibodies are the fundamental components of the immune system, and all classes of human antibodies are post-translationally modified by different kinds of glycans. The glycosylation of IgG has been well established to have a profound influence on its effector functions, changes in which will lead to serious diseases (Klasić and Zoldoš, 2021). Moreover, abnormal glycosylations of IgA have also been demonstrated to be associated with several diseases, and are promising targets and biomarkers for the future diagnosis and treatment of immune diseases and antitumor therapy.

### Immunoglobulin A nephropathy

Immunoglobulin A nephropathy (IgAN) is known to be the most common primary glomerulonephritis worldwide, which can lead to renal failure. IgAN is prominently characterized by the aberrant glycoform of IgA1, with deficient galactosylation of the terminal *O*-linked glycans in the IgA1 hinge region. Overexpression of this kind of Gal-deficient IgA1 (Gd-IgA1) can trigger the production of anti-glycan antibodies. And the high level of Gd-IgA1 in circulation can be recognized and bound by the autoantibodies of IgG or IgA to form immune complexes (Novak et al., 2018; Suzuki and Novak, 2021). Some of the immune complexes deposit in the glomerulus inducing the proliferation of mesangial cells. The subsequently release of pro-inflammatory cytokines such as TNF-α (Tumour Necrosis Factor alpha), IL-6 (Interleukin 6), and TGF-β (transforming growth factor-beta) by the mesangial cells can induce the inflammation and glomerulosclerosis and eventually cause renal injury.

The pathogenic mechanism for the generation of Gd-IgA1 is not yet clear. Previous studies have shown that the production of Gd-IgA1 can be affected by both genetic and behavioral factors. Kiryluk et al. (2017) have demonstrated that the C1GALT1 and C1GALT1C1 are the key genes encoding for galactosyltransferases in the biosynthesis of the *O*-glycosylation of IgA1, the mRNA levels of which determine the rate of Gd-IgA1 secretion in IgA1 producing cells. Meanwhile, Gale et al. (2017) have confirmed at the genetic level that the variation of C1GALT1 in different ethnic groups can affect the level of Gd-IgA1 in the circulation system. Moreover, the SNP (single nucleotide polymorphism, rs1047763) located in the core promoter region of the C1GALT1 gene is considered to be closely related to IgAN. This SNP is within the gene region bound to miRNA (miR-148b), the upregulation of which has been reported to be associated with the decreased expression of C1GALT1 and production of Gd-IgA1, so the SNP may affect the pathogenesis of IgAN by influencing the binding of miR-148b to C1GALT1. Other studies have shown that B lymphocytes can also affect IgA1 glycosylation by secreting different cytokines. It has been reported that cytokines such as IL-6 and IL-4 can promote Gd-IgA1 production by reducing the expression level of galactosyltransferase C1GALT1 or increasing the expression of sialyltransferase ST6GalNAc-II (Yamada et al., 2010; Suzuki et al., 2014). And compared to IL-4, IL-6 can enhance the aberrantly glycosylated IgA to a greater extent (Suzuki et al., 2014). Yamada et al. (2017) revealed that IL-6 can induce the enhanced and prolonged phosphorylation of STAT3 (signal transducer and activator of transcription 3), thus resulting in the overproduction of Gd-IgA1. Furthermore, Makita et al. (2020) indicated that Toll-like receptor 9 (TLR9) activation can improve the prodution of IL-6 and APRIL (A proliferation-inducing ligand). And IL-6 and APRIL can independently or synergisticly promote the synthesis of Gd-IgA1 and the corresponding immune complexes. These findings provide new insights into the future treatments of IgAN, such as genetic regulation and cytokines adjustment.

### Immunoglobulin A vasculitis (henoch-schonlein purpura)

IgA vasculitis (IgAV), formerly known as Henoch-Schonlein Purpura (HSP), is a kind of small-vessel vasculitis in children. It is often accompanied by complications such as gastrointestinal inflammation, arthritis and nephropathy. Approximately 40% of children with HSP will develop into nephritis (HSPN) (Pillebout and Sunderkötter, 2021). Similar to IgA nephropathy, the abnormal glycosylation of IgAl followed by deposition in the glomerular is a common feature in IgAV patients (Suzuki et al., 2018; Song et al., 2021). The deposition of the IgA1 immune complex can be found in the vascular wall, mucosal tissue, and glomerular mesangium of children, and the glycans appeared in the hinge regions of these deposited IgA1 are often accompanied by the loss of Gal residues (Song et al., 2021). It has been demonstrated that the abnormal *O*-glycan glycosylation of IgA1 in IgAN also appears in patients with HSP (Pillebout et al., 2017; Suzuki et al., 2018). Therefore, Gd-IgA1 is currently considered to play a key role in the pathogenesis of HSPN. Gd-IgA1 has been reported to be involved in mediating the inflammatory injury of HSP small vessels by activating cellular NF-κB and upregulating the expression of the inflammatory mediator IL-8, TNF-α and ICAM-1 (Ran and Ling, 2016). Besides Gal deficiency, Nakazawa et al. (2019) have demonstrated a decreased expression of GalNAc in the serum IgA1 of HSP patients through mass spectrometry analysis. Throughout the pathogenesis of IgAN and IgAV, it can be seen that the abnormally glycosylated IgA antibody cannot perform its normal function but will become the driving factor of immune diseases.

### Immunoglobulin A myeloma

IgA myeloma is a disease caused by the abnormal amplification of genes due to the uncontrolled division and proliferation of mutant IgA-producing cells (Wang et al., 2018). The concentration of serum IgA in patients with IgA myeloma can reach to more than 30 g/L. IgA myeloma is often accompanied by kidney, bone, and blood diseases, and other complications. Bosseboeuf et al. (2020) found that, in addition to the abnormal expression of IgA in these patients, the sialylation level of IgA was also reduced, affecting the binding of IgA to FcαRI. Renfrow et al. (2007) reported that the *O*-glycosylation sites in the hinge region of IgA1 is abnormal in these patients, which usually appear outside the common 3–6 glcosylation sites in that of healthy individuals. Furthermore, Suzuki et al. (2018) demonstrated that there was also a loss of Gal residue on the glycans of the IgA1 hinge region in the immune deposits of IgA myeloma patients complicated with nephropathy, which was similar to the Gd-IgA1 in patients with IgAN.

### Other inflammatory and autoimmune diseases

In addition to the above diseases, the alterations of IgA1 glycosylation are often accompanied by the development of Crohn’s disease and other autoimmune diseases such as systemic lupus erythematosus (SLE), rheumatoid arthritis (RA), Sjogren’s syndrome, celiac disease (Hansen et al., 2021). Although the pathogenesis of them remains unknown, it has been proposed that the presence of IgA autoantibodies, aberrant IgA glycosylation, or excessive IgA immune complexes can contribute to chronic inflammation and tissue damage through the effections of FcαRI-mediated immune responses (van Gool and van Egmond, 2020).

Crohn’s disease is generally referred to as an inflammatory bowel disease. At present, the pathogenesis of Crohn’s disease is not clear, and there are no corresponding serum markers for the detection. In glycosylation, the *N*-/*O*-linked glycans of serum IgA in patients and healthy volunteers have been compared (Inoue et al., 2012). Result showed that there was no difference in the *N*-linked glycans of IgA between the two groups, while the GalNAc modification on the *O*-linked glycans of IgA1 was significantly decreased in the patients, and strongly correlated with the severity of the disease. Therefore, the alteration of the GalNAc expression level in IgA1 *O*-linked glycans could be a promising diagnostic and prognostic marker for Crohn’s disease.

SLE is a chronic autoimmune disorder with injury in multiple organs, such as the skin, joints, kidney, and haematopoietic system. In SLE patients, a significant increase in serum IgA has been observed, and it can be 4–6 times higher than that of normal individuals (Matei and Matei, 2000). Moreover, the abnormal glycosylation has also been found in IgA1 isolated from female patients. The level of galactosylated glycans in the hinge region and the expression of unbisected biantennary as well as tri-, and tetraantennary glycans were shown to be decreased (Matei and Matei, 2000).

RA is one of the most commonly diagnosed autoimmune diseases. In addition to the well-known changes in IgG *N*-glycosylation, RA has also been shown to be associated with the alterations of IgA glycosylation. Wada et al. (2010) demonstrated the reduced GalNAc glycosylation in the IgA1 hinge region in the circulatory system of RA patients by mass spectrometry. Kratz et al. (2010) found that there were changes in Sia and Gal modifications on both IgG and IgA in the joint effusion of early RA patients. The terminal Sia on IgA decreased in the early stage and increased later, and terminal Gal decreased first and then returned to normal levels.

Primary Sjögren’s syndrome (pSS) is also a kind of autoimmune disease that mainly affects the exocrine glands of middle-aged women. In the serum of these patients, the *N*-glycans of IgA (especially for IgA1) has been found to be apparently oversialylated, while the galactosylation was reduced (Basset et al., 2000). In addition, in patients with celiac disease, an autoimmune gluten-sensitive enteropathy, the deficiency of galactosylation on IgA targeted or non-targeted with transglutaminase 2 (TG2) has also been found in their circulatory system (Lindfors et al., 2011).

### Immunoglobulin A glycosylation and cancer

A large number of studies have shown that glycosylation changes in human serum are a common phenomenon that can be observed in the occurrence and development of various cancers. In addition to the alteration in core glycans, the terminal glycan motifs such as high fucosylation and sialylation are often accompanied by malignant transformation (Jin et al., 2019). These tumor-associated carbohydrate antigens (TACAs) are immunogenic, and they can be recognized and combined by the autoimmuno antibodies and affect immunoregulation *in vivo*. Different from the bulk circulating antibodies, the glycoform of antigen-specific antibodies can be stimulated according to the different structures of TACAs and programmed by the immune system to drive unique effector functions (Commins, 2015).

In serum, the abnormal glycosylation of the IgA antibody corresponding to TACAs is expected to be an important biomarker for cancer diagnosis. It has been found that the aberrant glycosylation of IgA mainly appears in the serum of patients with colorectal and breast cancers, which provides an important theoretical basis for the early diagnosis of such diseases (Lomax-Browne et al., 2019; Zhang et al., 2019). The TACAs such as T, Tn, and sialyl-Tn can often be found in breast cancers patients. Among them, the Thomsen–Friedenreich antigen (TF or T antigen, Galβ1,3GalNAcα-O-Ser/Thr) is the most common. It is overexpressed on the cell surface of most tumors, e.g., breast, ovarian, prostate, and lung cancers, and is closely related to tumor development. In serum, increased sialylation at the overall level of the TF antigen-specific antibody is common in cancer, while in patients with breast cancer, the sialylation level of this antibody has been found to be reduced, and its sensitivity is increased, which shows its potential use as an antibody biomarkers in early breast cancer screening (Kurtenkov et al., 2018). Moreover, it has been reported that the abnormal *O*-glycosylated IgA1 carrying the Tn antigen (GalNAcα-O-Ser/Thr) exists in the invasive site of primary breast cancer cells, which is positively correlated with tumor metastasis (Welinder et al., 2013). Additionally, in the serum of colorectal cancer patients, the total level of IgA is not varied, whereas its reactivity against the RL2 *O*-GlcNAc antibody has been reported to be significantly increased compared to healthy individuals (Verathamjamras et al., 2021). These aberrant IgA glycosylations may provide valuable information for the future assessment and treatment of tumors.

## Glycoengineered immunoglobulin A antibodies for cancer immunotherapy

Antibody therapy is an effective method for the treatment of malignant diseases. At present, IgG antibodies are often used in cancer immunotherapy, but not all patients respond effectively to this treatment. Due to its unique immunomodulatory effect, IgA is regarded as a potential cancer immunotherapeutic antibody instead of IgG (Sterlin and Gorochov, 2021). IgA can activate neutrophils by binding to FcαRI and mediate their killing effect on tumor cells, such as ADCC (antibody-dependent cell cycotoxicity), cell phagocytosis, immune cell recruitment, the release of cytotoxic molecules, and cell necrosis. The tumor killing effect mediated by IgA in this way is much greater than that mediated by IgG (Brandsma et al., 2019). At present, IgA monoclonal antibodies targeting tumor cells such as breast cancer HER2 (human epidermal growth factor receptor 2) and B-cell lymphoma CD20 have shown a strong anti-tumor effect (Meyer et al., 2016). Moreover, the antibodies specific to both IgA and FcαRI can further activate the function of immune cells expressing FcαRI receptors, which can be developed as a new therapeutic drug for tumors (Breedveld and van Egmond, 2019).

It has been clearly demonstrated that the glycosylation of antibodies can affect their effector function and the finally therapeutic efficacy. Compared with IgG, IgA has more complex glycans and can be rapidly cleared by ASGPR, resulting in its short serum half-life and inconvenient production and purification process, which make the application of IgA antibodies in tumor therapy difficult. In order to improve the pharmacokinetic characteristics and *in vivo* anti-tumor effect of IgA, glycoengineered cell lines have been exploited to express specific recombinant IgA therapeutic antibodies with optimized glycosylation, e.g., with fewer glycosylation sites, a high degree of terminal sialylation degree, and reduced galactosylation (Lohse et al., 2016). In a mouse model, Rouwendal et al. (2016) reported that the highly sialyated N-glycans of anti-HER2 IgA slowed down tumor growth in FcαRI transgenic mice and prolonged the half-life of IgA antibodies. Several expression systems have been used for the production of IgA recombinant antibodies, such as plants (Larrick et al., 2001; Juarez et al., 2013), insect cells (Carayannopoulos et al., 1994), monkey-derived COS cells (Morton et al., 1993), baby hamaster kidney cells (Morton et al., 1993), myeloma cells (Yoo et al., 2010), and Chinese hamster ovary (CHO) cells (Pleass et al., 2003; Yoo et al., 2010; Moldt et al., 2014). In order to avoid the immune response caused by non-human glycan structures, such as α1-3-linked Gal and N-glycolylneuraminic acid (NeuGc) attached to IgA antibodies, human expression systems have also been constructed. Hart et al. (2017) have generated a panel of IgA antibodies targeting five different tumor antigens (MUC1, HER2, EGFR, TF, and CD20) by using human myelogenous leukemia-derived GlycoExpress (GEX) cell lines. All of the IgA antibodies expressed fully human glycans, and more than 60% of the N-glycans were sialylated, in contrast to only 20% sialylation for the IgA antibodies expressed in CHO cells. Moreover, the high-yield IgA antibodies produced by the human GEX expression system can bind to both specific antigens and FcαRI and can effectively inhibit the proliferation of cancer cells. These results further support the application prospect of IgA antibodies for cancer immunotherapy.

## Conclusion and outlook

In summary, IgA, as the most abundant immunoglobulins in the human body, has attracted much attention due to its biofunctionality and the diseases related to abnormal glycosylation. The aberrant glycoform of IgA has become a potential biomarker and a certain judgment basis for the clinical diagnosis of nephropathy, vasculitis, cancers, etc. Although some achievements have been made in the elucidation of IgA glycosylation structures, the pathogenic mechanism of IgA glycans involved in these diseases has not been identified. The *in vivo* studies on IgA is hampered due to the lack of suitable animal experimental models. The IgA expressed in species such as mice, rat, and rabbits are quite different from humans in its subtypes, glycosylations, forms and distributions. Especially, these experimental species do not have human IgA1 subclass and mice do not even express FcαRI (Snoeck et al., 2006). Such species differences constrain the research on human IgA as well as its glycosylation function. Furthermore, as a promising immunotherapeutic antibody, there are still some problems that need to be solved in the production and application of IgA antibodies due to their high heterogeneity in *N*- and *O*-glycan modifications, their short serum half-life, and so on. To date, no IgA antibody has been used in clinical trials. Therefore, in-depth studies on the IgA glycosylation and its immune effector mechanism are still needed in the future. Moreover, new strategies need to be further explored to produce recombinant IgA antibodies with consistent glycosylation, so that they can be used in clinical treatment. IgA antibodies are also expected to be combined with IgG to develop homotypic cross-antibodies containing both IgG and IgA Fc domain residues, so that they can not only activate neutrophils- and macrophage-mediated tumor killing but also play a role in complement-dependent cytotoxicity, and their kinetic characteristics can be improved additionally.

## Glossary

IgA: Immunoglobulin A
FcαRI: Fc alpha Receptor I
IgAN: IgA nephropathy
IgAV: IgA vasculitis
SLE: systemic lupus erythematosus
RA: rheumatoid arthritis
H/L chain: heavy/light chain
Fab: antigen-binding fragment
Fc: crystallizable fragment
VH/L: varible domain on the heavy/light chain
CH/L: constant domain on the heavy/light chain
mIgA: monomeric IgA
pIgA: polymeric IgA
ITAM/ITIM: Immunoreceptor tyrosine-based activation/inhibition motif
SIgA: secretory immunoglobulin A
dIgA: dimer IgA
pIgR: polymeric immunoglobulin receptor
SC: secretory component
Asn: Asparagine
Ser/Thr: Serine/Threonine
Gal: galactose
GalNAc: N-acetylgalactosamine
Glc: glucose
Fuc: fucose
NeuAc: N-acetylneuraminic acid
Sia: sialic acid
MBL/MBP: mannose-binding lectins/proteins
FcαRI: Fcα-receptor I
FcRL4: Fc receptor-like 4
ASGPR: asialoglycoprotein receptor
Fcα/μR: Fcα/μ receptor
ASGPR: asialoglycoprotein receptor
NET: neutrophil extracellular traps
IVA: influenza A virus
HA: hemagglutinin
HIV: human immunodeficiency virus
SARS-CoV-2: syndrome coronavirus 2
PNG: peptidoglycans
LPS: lipopolysaccharide
B. theta: Bacteroides thetaiotaomicron
LPS: lipopolysaccharide
Gd-IgA: Gal-deficient IgA1
TNF-α: Tumour Necrosis Factor alpha
IL-6: Interleukin 6
TGF-β: transforming growth factor-beta
SNP: single nucleotide polymorphism
STAT3: signal transducer and activator of transcription 3
TLR9: Toll-like receptor 9
APRIL: A proliferation inducing ligand
pSS: Primary Sjögren’s syndrome
TACAs: tumor-associated carbohydrate antigens
HER2: human epidermal growth factor receptor 2
EGFR: epidermal growth factor receptor
MUC1: Mucin 1
TF: Transferrin
ADCC: antibody-dependent cell cycotoxicity
